# Utility of the LACE index to assess risk of mortality and readmission in patients with spinal infections

**DOI:** 10.1007/s10143-024-02411-2

**Published:** 2024-04-17

**Authors:** Ralph T. Schär, Mattia Branca, Andreas Raabe, C. Marvin Jesse

**Affiliations:** 1 Department of Neurosurgery, Inselspital, Bern University Hospital, University of Bern, Freiburgstrasse 18, Bern, 3010 Switzerland; 2 Clinical Trials Unit Bern, University of Bern, Bern, Switzerland

**Keywords:** Hospital readmission, LACE index, Logistic regression, Mortality, Odds ratio, Outcomes, Preoperative risk assessment, Risk assessment, Spinal infections, Spine surgery

## Abstract

Retrospective cohort study. To assess the utility of the LACE index for predicting death and readmission in patients with spinal infections (SI). SIs are severe conditions, and their incidence has increased in recent years. The LACE (Length of stay, Acuity of admission, Comorbidities, Emergency department visits) index quantifies the risk of mortality or unplanned readmission. It has not yet been validated for SIs. LACE indices were calculated for all adult patients who underwent surgery for spinal infection between 2012 and 2021. Data were collected from a single academic teaching hospital. Outcome measures included the LACE index, mortality, and readmission rate within 30 and 90 days. In total, 164 patients were analyzed. Mean age was 64.6 (± 15.1) years, 73 (45%) were female. Ten (6.1%) patients died within 30 days and 16 (9.8%) died within 90 days after discharge. Mean LACE indices were 13.4 (± 3.6) and 13.8 (± 3.0) for the deceased patients, compared to 11.0 (± 2.8) and 10.8 (± 2.8) for surviving patients (*p* = 0.01, *p* < 0.001), respectively. Thirty-seven (22.6%) patients were readmitted ≤ 30 days and 48 (29.3%) were readmitted ≤ 90 days. Readmitted patients had a significantly higher mean LACE index compared to non-readmitted patients (12.9 ± 2.1 vs. 10.6 ± 2.9, < 0.001 and 12.8 ± 2.3 vs. 10.4 ± 2.8, *p* < 0.001, respectively). ROC analysis for either death or readmission within 30 days estimated a cut-off LACE index of 12.0 points (area under the curve [AUC] 95% CI, 0.757 [0.681–0.833]) with a sensitivity of 70% and specificity of 69%. Patients with SI had high LACE indices that were associated with high mortality and readmission rates. The LACE index can be applied to this patient population to predict the risk of early death or unplanned readmission.

## Key Points

- Patients with surgically managed spinal infections had high 30-day readmission (22.6%) and mortality (6.1%) rates, which corresponds well with high-risk LACE index scores.
- Close postoperative monitoring of these patients is critical.
- The LACE index may serve as a useful predictive tool for identifying patients with higher risks of early death or unplanned readmission after discharge from hospital.

## Introduction

Spinal infections (SI) are feared and potentially life-threatening conditions, and their incidence has been increasing in recent years [1]. SI can arise primarily through hematogenous spread, or secondarily following spine surgery or spinal interventions leading to surgical site infections (SSI). SSIs can lead to significant morbidity and it has been shown that SSIs are associated with increased mortality after elective spine surgery [2]. 

Patients with SI are often of older age and burdened by distinct comorbidities. When managed surgically, given the increased morbidity and cost associated with perioperative complications and unplanned readmissions, the accurate risk stratification of patients with SI is of great clinical value. In recent years, in an attempt to increase healthcare value and lower healthcare related costs, much effort has been invested in understanding and predicting the disease-related risks of mortality and unplanned hospital readmissions [3]. 

The LACE (an acronym for Length of stay, Acuity of admission, Charlson Comorbidity Index [CCI] score, and Emergency department visits within the previous 6 months) index was first introduced in 2010 by van Walraven and colleagues [4]. It was developed as a tool and composite score to predict the risk of mortality or unplanned 30-day hospital readmission after inpatient hospital discharge. The LACE index is a scoring system that ranges from 1 to 19. Scores of 1 to 4 are considered low risk for early mortality or 30-day readmission, 5 to 9 are moderate risk, and scores greater than 9 are defined as high risk. Studies have shown that a score of 10 or higher is most predictive of poor outcomes following hospital discharge [5, 6]. While the LACE index has been validated in large patient populations in general medicine, little is known about its utility in spine surgery, let alone for patients with SI. Given the high morbidity and increased mortality of spinal infections, it is essential for spine surgeons and other physicians treating these patients to better understand and predict the risk of poor outcomes after discharge from hospital and readmissions in this patient population.

The aim of this study was to assess the utility of the LACE index with regard to risk of mortality and unplanned readmission within 30 days after hospital discharge in a cohort of patients with surgically managed SI.

## Materials and methods

### Study design and patient selection

We performed a retrospective cohort study and analysis of all adult patients who underwent surgical treatment for primary (de novo infections through hematogenous spread) or secondary spinal infections (SSIs) at our neurosurgical department situated in an academic teaching hospital between 2012 and 2021. All data were documented according to institutional standards, including principles of good clinical practice (GCP). This study was approved by the local ethics committee of the Canton of Bern, Switzerland (2023 − 00461). All the patients included in this study followed the general consent procedure, permitting the use of health-related data.

### LACE index

The variables necessary to compute the LACE index included length of stay in days (“L”), acuity of patient admission (“A”), comorbidity of the patient (measured with the Charlson comorbidity index score) (“C”), and emergency department (ED) use (number of visits to the ED in the six months before admission). The LACE index was computed for all the patients. Data were collected from our hospital’s electronic health information system with access to all ED admissions. Patients who died during their hospital stay were excluded from the analysis, as a LACE index value is generated for each patient at discharge. We chose to use the original LACE index as opposed to the LACE + score, which was later developed by the same authors as an extension of the LACE index but was intended to be used with administrative data only. Both scores have been shown to predict 30-day readmission or death with excellent calibration and good discrimination [7]. 

### Statistical analysis

Demographic and clinical data were collected using our electronic patient and clinical information system. Descriptive statistics were performed using frequencies and percentages for categorical variables, and means with standard deviations for continuous variables. Parametric and/or non-parametric tests, Student’s t-test, Wilcoxon-Mann-Whitney U-test for continuous variables, and Chi-squared test for categorical variables were performed to descriptively compare the patient’s characteristics and LACE score variables. ROC analysis was performed to estimate the area under the curve (AUC) of the score to discriminate between patients with and without the outcome, and the estimated cut-off was extracted for the available sample based on the Youden Index, which maximizes the sum of the sensitivity and specificity. Univariate logistic regression for the baseline variables was performed to compare the association between them and different outcomes. Furthermore, a multivariable logistic regression was performed with all variables with a p-value lower than 0.10 and where the outcome had at least 15–20 events to compare the crude and adjusted associations. Stata 17.0 and R 4.2.1 software were used for statistical analysis.

## Results

### Patients’ characteristics

Of the 167 patients treated for SI, three died during their hospital stay and were excluded from the final analysis. Thus, 164 patients were included and analyzed in this study. The mean age was 64.6 (± 15.1) years, and 73 (45%) were female. The mean LACE index score for all patients was 11.1 (± 2.9). Secondary SI was slightly more frequent than primary SI (57% vs. 43%), and the lumbar spine was the most frequent SI site, accounting for 51% of all cases. A detailed overview of the patients’ characteristics and clinical data, including a breakdown of the LACE components, body mass index (BMI), American Society of Anesthesiologists Physical Status (ASA) scores, as well as etiology (primary or secondary) and location of spinal infection, are outlined in Table 1.

**Table 1:** Overview of patients’ characteristics

Characteristic	Total (*n* = 164)
Age (years), mean (±SD)	64.6 (±15.1)
Sex - n (%)	
Female	73 (45%)
Male	91 (55%)
BMI (kg/m2), mean (±SD)	27.3 (±6.2)
ASA score, mean (±SD)	3.0 (±0.7)
CRP at diagnosis (mg/L), median	29.1 (7.2, 108.0)
Etiology - n (%)	
Primary	70 (43%)
Secondary	94 (57%)
Location - n (%)	
Cervical	27 (16%)
Thoracic	27 (16%)
Lumbar	83 (51%)
Multilocular	27 (16%)
LACE index components	
Length of stay (days), mean (±SD)	19.9 (14.8)
Acuity of admission, n (%)	
No	12 (7.3%)
Yes	152 (92.7%)
CCI, mean (±SD)	2.0 (2.1)
ED visits < 6 months, mean (±SD)	0.5 (0.8)
LACE Index, mean (±SD)	11.1 (2.9)

Data are presented as mean ± standard deviation or median with IQR for continuous variables and frequency with percentages for categorical variables. BMI, body mass index; CCI, Charlson comorbidity index; ED, emergency department; SD, standard deviation

### Mortality rates

**Fig. 1:**
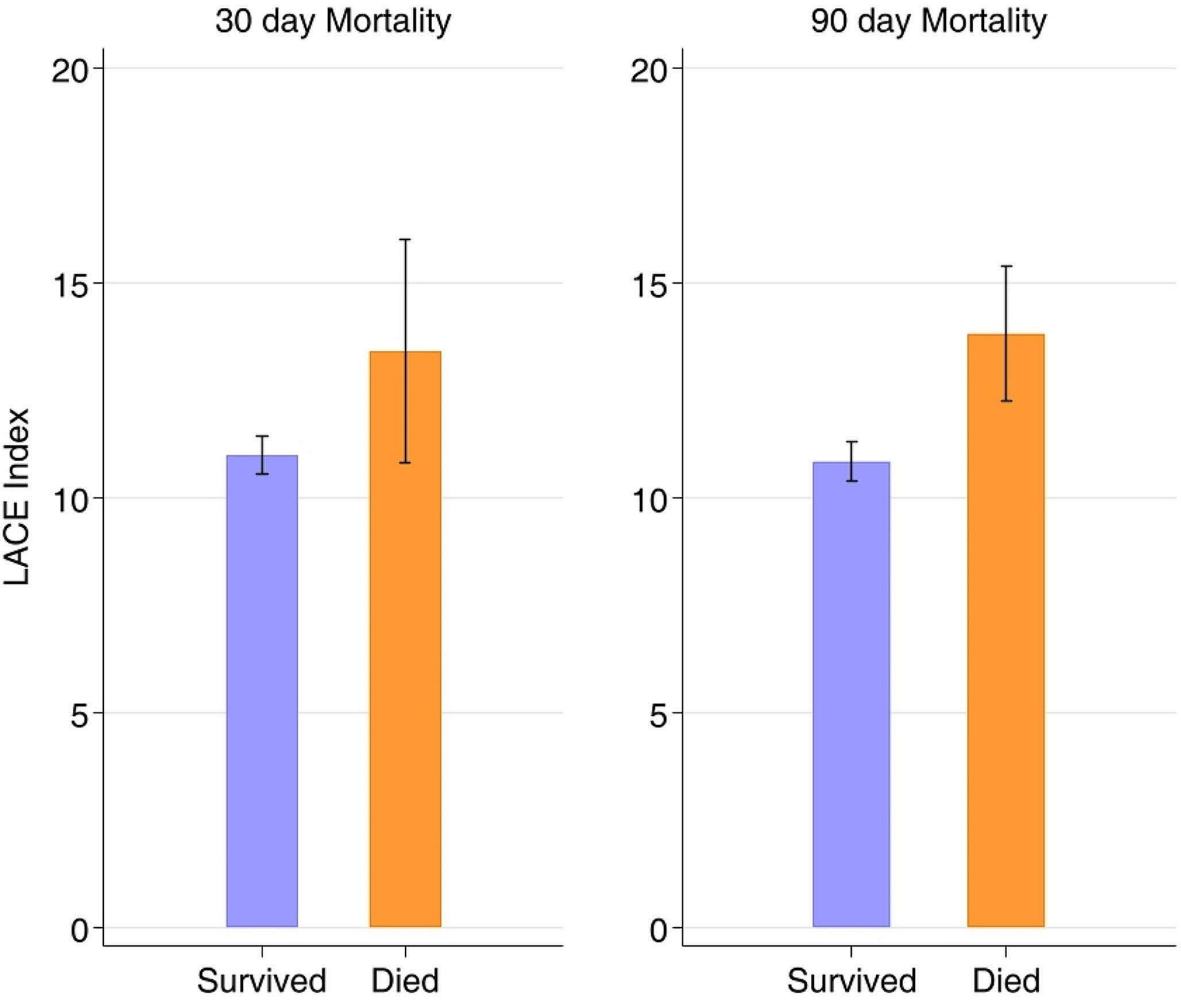
Correlation between the LACE index and mortality

Ten of the 164 patients died within 30 days, and 16 died within 90 days after hospital discharge, corresponding to 30-day and 90-day mortality rates of 6.1% and 9.8%, respectively. Patients who died within 30 days had significantly higher mean LACE indices than surviving patients (13.4 ± 3.6 vs. 11.0 ± 2.8, 95%CI -2.43 [-4.26 to -0.58], *p* = 0.01). This was also observed in patients who died within 90 days (13.8 ± 3.0 vs. 10.8 ± 2.8, 95%CI -2.97 [-4.42, -1.53], *p* < 0.001) (Table 2). These outcomes are shown in Fig. 1.

**Table 2:** Breakdown of LACE index components for death, readmission and death or readmission within 30 days and 90 days

	**Death ≤ 30 days**				**Death ≤ 90 days**			
	**No (** ***n*** ** = 154)**	**Yes (** ***n*** ** = 10)**	**Mean or risk difference (95%-CI)**	***P*** **value**	**No (** ***n*** ** = 148)**	**Yes (** *n* ** = 16)**	**Mean or risk difference (95%-CI)**	***P*** **value**
**LACE Index**, mean (± SD)	10.98 (±2.80)	13.40 (±3.63)	-2.43 (-4.26 to -0.58)	0.010	10.84 (2.76)	13.81 (2.97)	-2.97 (-4.42 to -1.53)	< 0.001
**L**ength of stay (days), mean (± SD)	19.79 (±15.08)	21.40 (±11.22)	-1.61 (-11.20 to 7.99)		19.11 (14.64)	27.13 (15.25)	-8.02 (-15.66 to -0.38)	
**A**cuity of admission - n yes (%)	142 (92.2%)	10 (100%)	-0.08 (-0.12 to -0.04)		136 (91.89%)	16 (100%)	-0.08 (-0.13 to -0.04)	
**C**CI – age, mean (± SD)	1.88 (2.06)	3.50 (2.72)	-1.62 (-2.97 to -0.26)		1.80 (2.03)	3.69 (2.41)	-1.89 (-2.96 to -0.82)	
**E**D visits < 6 months, mean (± SD)	0.45 (±0.73)	1.10 (±1.20)	-0.65 (-1.14 to -0.15)		0.43 (0.73)	1.06 (1.00)	-0.63 (-1.03 to -0.24)	
	**Readmission ≤ 30 days**				**Readmission ≤ 90 days**			
	**no (** ***n*** ** = 127)**	**yes (** ***n*** ** = 37)**	**Mean or risk difference (95%-CI)**	***P*** **value**	**no (** ***n*** ** = 127)**	**yes (** ***n*** ** = 37)**	**Mean or risk difference (95%-CI)**	***P*** **value**
**LACE Index**, mean (± SD)	10.62 (2.91)	12.86 (2.12)	-2.24 (-3.26 to -1.23)	< 0.001	10.44 (2.85)	12.79 (2.32)	-2.35 (-3.27 to -1.43)	< 0.001
**L**ength of stay (days), mean (± SD)	18.97 (14.79)	23.05 (14.79)	-4.09 (-9.54 to 1.37)		18.16 (14.52)	24.08 (14.94)	-5.93 (-10.89 to -0.97)	
**A**cuity of admission - n yes (%)	115 (90.55%)	37 (100%)	-0.09 (-0.15 to -0.04)		104 (89.66%)	48 (100%)	-0.10 (-0.16 to -0.05)	
**C**CI – age, mean (± SD)	1.87 (2.16)	2.35 (2.02)	-0.48 (-1.26 to 0.31)		1.81 (2.12)	2.40 (2.14)	-0.59 (-1.31 to 0.13)	
**E**D visits < 6 months, mean (± SD)	0.33 (0.69)	1.03 (0.83)	-0.69 (-0.96 to -0.43)		0.28 (0.64)	1.00 (0.85)	-0.72 (-0.96 to -0.48)	
	**Death or readmission ≤ 30 days**				**Death or readmission ≤ 90 days**			
	**no (** ***n*** ** = 118)**	**yes (** ***n*** ** = 46)**	**Mean or risk difference (95%-CI)**	***P*** **value**	**no (** ***n*** ** = 104)**	**yes (** ***n*** ** = 60)**	**Mean or risk difference (95%-CI)**	***P*** **value**
**LACE Index**, mean (± SD)	10.40 (2.73)	13.00 (2.50)	-2.60 (-3.52 to -1.69)	< 0.001	10.03 (2.49)	13.03 (2.58)	-3.00 (-3.81 to -2.20)	< 0.001
**L**ength of stay (days), mean (± SD)	18.73 (15.02)	22.87 (14.12)	-4.14 (-9.21 to 0.93)		17.05 (13.99)	24.82 (15.11)	-7.77 (-12.38 to -3.16)	
**A**cuity of admission - n yes (%)	106 (89.83%)	46 (100.00%)	-0.10 (-0.16 to -0.05)		92 (88.46%)	60 (100%)	-0.12 (-0.18 to -0.05)	
**C**CI – age, mean (± SD)	1.73 (2.06)	2.63 (2.21)	-0.90 (-1.62 to -0.18)		1.54 (1.89)	2.75 (2.33)	-1.21 (-1.87 to -0.55)	
**E**D visits < 6 months, mean (± SD)	0.27 (0.60)	1.04 (0.92)	-0.77 (-1.01 to -0.53)		0.18 (0.50)	1.02 (0.89)	-0.83 (-1.05 to -0.62)	

Differences between groups were compared using the t-test for continuous variables and the Pearson Chi-2 for binary variables. The effect measures displayed are the mean difference with 95%CI for the continuous variables and risk difference with 95%CI for binary variables. P values were only computed for the LACE index. CCI, Charlson comorbidity index; ED, emergency department; SD, standard deviation

### Readmission rates

Thirty-seven (22.6%) patients were readmitted within 30 days, and 48 (29.3%) were readmitted within 90 days of hospital discharge. All readmitted patients had significantly higher mean LACE indices than non-readmitted patients (within 30 days: 12.9 ± 2.1 vs. 10.6 ± 2.9, 95% CI -2.24 [-3.26 to -1.23], < 0.001; within 90 days: 12.8 ± 2.3 vs. 10.4 ± 2.8, 95%CI -2.35 [-3.27 to -1.43], *p* < 0.001) (Table 2).

### Logistic regression analysis

Univariate logistic regression showed associations between both age (*p* = 0.029) and LACE index (*p* = 0.014) and the risk of death within 30 days of hospital discharge. Analysis of the ASA scores revealed an association for readmission within 30 days (*p* = 0.037) and for death or readmission within 30 days (*p* = 0.016). Of all the tested variables, the LACE index remained the only significant surrogate parameter for all calculations analyzing readmission and mortality within 30 and 90 days (Table 3).

**Table 3:** Univariable and multivariable logistic regression to analyze readmission and mortality within 30 and 90 days of discharge

	Death ≤ 30 days (*n* = 10/164)		Readmission ≤ 30 days (*n* = 37/164)		Death or readmission ≤ 30 days (*n* = 46/164)		Death ≤ 90 days (*n* = 16/164)		Readmission ≤ 90 days (*n* = 48/164)		Death or readmission ≤ 90 days (*n* = 60/164)	
Univariable logistic regression	OR (95%-CI)	P value	OR (95%-CI)	P value	OR (95%-CI)	P value	OR (95%-CI)	P value	OR (95%-CI)	P value	OR (95%-CI)	P value
Age	1.07 (1.01–1.14)	0.029	1.03 (1.00-1.06)	0.033	1.04 (1.01–1.07)	0.004	1.04 (1.00-1.09)	0.059	1.04 (1.01–1.06)	0.009	1.04 (1.02–1.07)	0.001
Sex	3.42 (0.70-16.64)	0.127	1.43 (0.67–3.02)	0.354	2.00 (0.98–4.08)	0.058	2.62 (0.81–8.50)	0.109	1.18 (0.60–2.33)	0.637	1.49 (0.78–2.85)	0.228
BMI (kg/m2)	1.02 (0.93–1.13)	0.632	1.00 (0.94–1.06)	0.987	1.00 (0.95–1.06)	0.881	0.96 (0.87–1.05)	0.331	1.00 (0.95–1.06)	0.995	0.99 (0.94–1.04)	0.729
ASA score	2.22 (0.74–6.68)	0.155	1.95 (1.04–3.64)	0.037	2.07 (1.15–3.73)	0.016	1.61 (0.69–3.79)	0.272	1.70 (0.97–2.98)	0.062	1.69 (1.00-2.85)	0.051
CRP at diagnosis (mg/L)*	1.16 (0.79–1.71)	0.449	1.05 (0.85–1.29)	0.677	1.08 (0.89–1.32)	0.432	1.16 (0.85–1.58)	0.359	1.00 (0.83–1.21)	0.988	1.06 (0.89–1.27)	0.522
Etiology	0.30 (0.07–1.19)	0.087	1.30 (0.61–2.75)	0.499	0.96 (0.48–1.90)	0.898	0.55 (0.19–1.54)	0.253	1.74 (0.86–3.52)	0.121	1.48 (0.77–2.84)	0.238
Thoracic vs. cervical	1.00 (0.18–5.46)	1.000	1.00 (0.28–3.61)	1.000	0.84 (0.27–2.66)	0.770	1.31 (0.31–5.51)	0.715	1.19 (0.38–3.75)	0.770	1.16 (0.40–3.43)	0.783
Lumbar vs. cervical	0.30 (0.06–1.58)	0.156	1.11 (0.39–3.14)	0.842	0.77 (0.30–1.95)	0.577	0.37 (0.09–1.49)	0.161	1.02 (0.40–2.65)	0.961	0.78 (0.32–1.90)	0.587
Multilocular vs. cervical	0.31 (0.03–3.16)	0.321	0.80 (0.21-3.00)	0.736	0.57 (0.17–1.92)	0.365	0.46 (0.08–2.75)	0.395	0.68 (0.20–2.31)	0.536	0.61 (0.20–1.89)	0.394
LACE Index	1.34 (1.06–1.69)	0.014	1.34 (1.16–1.54)	< 0.001	1.43 (1.23–1.66)	< 0.001	1.47 (1.19–1.80)	< 0.001	1.37 (1.19–1.57)	< 0.001	1.59 (1.35–1.86)	< 0.001
**Multivariable logistic regression**	**OR (95%-CI)**	***P*** **value**	**OR (95%-CI)**	***P*** **value**	**OR (95%-CI)**	***P*** **value**	**OR (95%-CI)**	***P*** **value**	**OR (95%-CI)**	***P*** **value**	**OR (95%-CI)**	***P*** **value**
Age			1.02 (0.99–1.05)	0.150	1.03 (1.00-1.07)	0.029	1.04 (0.99–1.09)	0.127	1.03 (1.00-1.06)	0.055	1.04 (1.01–1.07)	0.015
Sex					1.26 (0.56–2.83)	0.578						
ASA score			1.49 (0.73–3.05)	0.268	1.48 (0.74–2.98)	0.269			1.19 (0.62–2.29)	0.599	0.98 (0.51–1.89)	0.949
LACE Index			1.30 (1.12–1.51)	0.001	1.38 (1.18–1.61)	< 0.001	1.46 (1.18–1.81)	0.001	1.34 (1.16–1.55)	< 0.001	1.57 (1.33–1.86)	< 0.001

*Based on the natural logarithm of CRP values; for multivariable logistic regression, only variables with p = < 0.1 are included; ASA, American Society of Anesthesiologists’ classification of Physical Health; BMI, body mass index; CI, confidence interval; CRP, C-reactive protein; OR, odds ratio

### ROC analysis

For death within 30 days, ROC analysis estimated a cut-off LACE index of ≥ 15.0 points (AUC 95% CI of 0.684 [0.484–0.884]) with a sensitivity of 50% and specificity of 86%. The same analysis for either death or readmission within 30 days estimated a cut-off LACE index of 12.0 points (AUC 95% CI of 0.757 (0.681–0.833)) with a sensitivity of 70% and specificity of 69%. The estimated cut-off LACE indices for both readmission within 30 and 90 days were also ≥ 12.0 points. All the ROC analysis results are presented in Table 4.

**Table 4:** ROC Analysis

Outcome	AUC (95%-CI)	Estimated cut-off point (≥)	Sensitivity and Specificity
Death within **≤** 30 days	0.684 (0.484 to 0.884)	15.0	50%; 86%
Death or readmission **≤** 30 days	0.757 (0.681 to 0.833)	12.0	70%; 69%
Death **≤** 90 days	0.764 (0.630 to 0.898)	13.0	69%; 74%
Readmission **≤** 90 days	0.747 (0.670 to 0.823)	12.0	71%; 70%

AUC, area under the curve with 95%-CI and estimated cut-off point based on the Youden Index that maximizes the sum of the sensitivity and specificity

## Discussion

This is the first study to evaluate the utility of the LACE index in patients with SI. Herein, we describe the utility of the LACE index in patients with primary or secondary SI who underwent surgical management. Spinal infections are feared and life-threatening conditions with reported mortality rates of up to 20%.^1^ In our cohort of 164 patients with SI who were managed surgically, 30-day mortality rate was as high as 6%. When including the rate of unplanned hospital readmission to mortality within 30 days, the adverse event rate increased to 28%. Our results showed a good association between mortality and readmission rates, with higher LACE indices for these adverse events. Logistic regression analysis indicated that patients with a higher LACE index had significantly higher rates of readmission and mortality. The ASA Physical Status class remains one of the most widely used risk-stratification metrics for medical complications and mortality after surgery, and it also showed a good association with higher mortality within 30 days in our present study [8]. However, in our patient cohort, the LACE index clearly outperformed the ASA score as a predictor of mortality and readmission based on logistic regression analysis. Furthermore, our analysis showed that for patients with a LACE index of ≥ 12 points, the sensitivity and specificity for either death or readmission within 30 days following hospital discharge were 70% and 69%, respectively.

As with any surgical specialty, it is crucial to assess patient outcomes including mortality and readmission rates. This is especially true for the surgical management of patients with SI since complication and mortality rates are known to be relatively high, as mentioned above. In addition, patients with SI are at high risk for perioperative complications and have higher morbidity and unplanned readmission rates [9]. For this patient population, accurate prediction of a patient’s individual risk profile regarding readmission and mortality is of utmost importance and value to allow for enhanced preoperative patient evaluation, risk stratification, and postoperative monitoring. However, a LACE index is generated for a patient only after discharge from hospital. Evidently, this limits its utility during acute care in hospital. Still, as healthcare professionals and policymakers alike strive to contain healthcare costs, accurate prediction of mortality after discharge from hospital and hospital readmission has become increasingly important. Therefore, reducing hospital readmission rates has long become a clinical and policy priority. In addition, readmission following surgery has gained increasing attention as a performance measure [10]. In this regard, the LACE index may serve as an easy-to-use tool in clinical practice and for hospital administrators alike.

In recent years, the LACE index has gained attention as a promising tool for predicting the likelihood of adverse events following surgery [11, 12]. Regarding outcome prediction, age, insurance status, paralysis, and medical comorbidities are thought to be possible predictors of morbidity, mortality, and expense of care for patients following surgical treatment of spinal epidural abscess [13]. In contrast to widely used traditional statistical approaches, recent studies have reported on machine learning based models for prediction of major perioperative complications and 30-day readmission after anterior cervical fusion surgery, or for readmission and estimated cost savings for patients undergoing posterior lumbar fusion surgery [14, 15]. Interestingly, despite acknowledging the discriminatory ability of the LACE index, Rezaii and colleagues maintained superior predictive ability of their machine learning model compared to the LACE index in predicting readmissions and projected cost reductions based on their data [1, 14]. However, these machine learning algorithms lack external validation and are therefore not yet fit for clinical implementation. In 2022, an assessment score for the preoperative estimation of mortality to support decision-making in the treatment of SI was published by Lener and colleagues [1]. 

The LACE index, although it accounts for only four general risk domains, seems to provide a sufficiently comprehensive assessment of patient risk after hospital discharge. It can therefore help provide guidance to clinicians and surgeons in making informed decisions. By quantifying patient risk, surgeons can more accurately determine the most appropriate postacute care management, and effectively allocate resources.

### Limitations, generalizability

Our study has several limitations. First, our analysis is limited by the retrospective nature of data collection. Therefore, there is an inherent risk of underreporting the actual rate of adverse events or other data inaccuracies. As such, it is possible that not all deaths were captured and accounted for in our hospital record system. Second, the generalizability of our analysis is limited by the distinct pathology of surgically managed spinal infections in adult patients. In addition, as we present data from a single, tertiary care, university-affiliated teaching hospital, our results may not be generalizable to the broader spine surgery community, as SI patients are often critically ill and require highly specialized multidisciplinary treatments. Finally, the LACE index was originally developed primarily for medical patients. For this reason, factors specific to spine surgery, such as surgical techniques and approaches, intraoperative complications, and specific comorbidities associated with spinal disorders, were not accounted for.

## Conclusions

Patients with surgically managed spinal infections had high-risk LACE indices that correlated well with both high mortality and readmission rates. The LACE index may serve as a useful tool to predict the risk of early death or unplanned readmission of patients with surgically managed spinal infections after hospital discharge.

## Data Availability

The data that support the findings of this study are available on request from the corresponding author (RTS).
